# Expression of long noncoding RNA MALAT1 correlates with increased levels of Nischarin and inhibits oncogenic cell functions in breast cancer

**DOI:** 10.1371/journal.pone.0198945

**Published:** 2018-06-18

**Authors:** Steven C. Eastlack, Shengli Dong, Yin Y. Mo, Suresh K. Alahari

**Affiliations:** 1 Department of Biochemistry and Molecular Biology, Stanley S. Scott Cancer Center, LSUHSC School of Medicine, New Orleans, LA, United States of America; 2 Department of Pharmacology and Toxicology, University of Mississippi Medical Center, Jackson, MS, United States of America; University of South Alabama Mitchell Cancer Institute, UNITED STATES

## Abstract

Malat1 is a long noncoding RNA with a wide array of functions, including roles in regulating cancer cell migration and metastasis. However, the nature of its involvement in control of these oncogenic processes is incompletely understood. In the present study, we investigate the role of Malat1 and the effects of Malat1 KO in a breast cancer cell model. Our selection of Malat1 as the subject of inquiry followed initial screening experiments seeking to identify lncRNAs which are altered in the presence or absence of Nischarin, a gene of interest previously discovered by our lab. Nischarin is a well characterized tumor suppressor protein and actively represses cell proliferation, migration, and invasion in breast cancer. Our microarray screen for lncRNAs revealed multiple lncRNAs to be significantly elevated in cells ectopically expressing Nischarin compared to control cancer cells, which have only marginal Nisch expression. Using these cells, we assess how the link between Nischarin and Malat1 affects cancer cell function, finding that Malat1 confers an inhibitory effect on cell growth and migration which is lost following Malat1 KO, but in a Nisch-dependent context. Specifically, Malat1 KO in the background of low Nischarin expression had a limited effect on cell functions, while Malat1 KO in cells with high levels of Nischarin led to significant increases in cell proliferation and migration. In summary, this project provides further clarity concerning the function of Malat1, specifically in breast cancer, while also indicating that the Nischarin expression context is an important factor in the determining how Malat1 activity is governed in breast cancer.

## Introduction

Long noncoding RNAs (lncRNAs) are a distinct subclass of noncoding RNAs defined as being longer than 200 nucleotides in length and were first described in 2002 [1]. As a group, noncoding RNAs have received growing interest in recent years. The role of these long regions of noncoding RNAs was clandestine for decades but recent evidence has uncovered that these molecules exert a multitude of effects within cells, such as regulation of transcription, control of posttranscriptional functions (*e*.*g*. splicing, translation) regulation of small RNA function, and more [2,3]This has ignited considerable interest into a group of molecules that was historically thought to be nonfunctional artifacts since they do not code for a protein product. Indeed, their importance is further highlighted by the awareness that of lncRNA genetic elements far outnumber protein-coding RNAs, which estimates as high 70–90% of the genome giving rise to lncRNA at some point during embryonic development [4].

Malat1 (Metastasis-associated lung adenocarcinoma transcript 1), also called NEAT2, is a lncRNAs discovered in the context of lung cancer and observed to exert a pro-metastatic effect [5]. In such cases it has also been found to function as a prognostic factor for metastasis and patient survival in both squamous and nonsquamous lung adenocarcinomas [6,7]. Later studies have reported it to be overexpressed in other carcinomas, such as breast, colon, prostate and liver [8]. Mechanistically, Malat1 appears to regulate gene expression, which results in the effects on cell migration and metastasis in cancer [9,10]. In breast cancer, Malat1 has alternatively been described as either a tumor suppressor or an oncogene. One recent study observed that knockdown of Malat1 could enhance migration and invasion in breast cancer cells [11], while a subsequent study reported the opposite outcome, specifically, Malat1 overexpression could induce migration and invasion in breast cancer cells [12]. Thus, the precise nature and function of this lncRNAs in the context of breast cancer is in need of continued investigation.

In the present study, we investigate the role of Malat1 in breast cancer in the expression background of the gene Nischarin, a protein previously characterized by our lab [13]. Nischarin is a tumor suppressor which is frequently underexpressed in breast cancer. Our initial screens of breast cancer MDA-MB-231 cells showed that Malat1 expression was positively correlated with the level of Nischarin present in the cell. In MDA-MB-231 cells, however, Nischarin expression is limited. We previously generated 231 cells which ectopically express the Nisch gene [14].Using both the 231 wild type and 231-Nisch cells, we sought to knockout (KO) Malat1 using a CRISPR-cas9 system and observed the effects. We found that, like Nischarin, Malat1 expression led to reduced cell proliferation and migration. The KO of Malat1 resulted in the enhancement of migration and proliferation, but this result was found to occur in a Nischarin-dependent context. Namely, in cells with low levels of Nischarin, the KO of Malat1 resulted in little change in cell behavior, while in cells overexpressing Nischarin, the KO of Malat1 had a significant effect, and led to the restoration of cell proliferative and migratory capacity. We suspect this finding is due to the correlated nature of Nisch and Malat1 expression—i.e., in Nischarin-low cells, Malat1 expression is also low, and thus KO of this lncRNA has little consequence. Conversely, Nischarin-high cells harbor higher levels of Malat1 expression, and thus the effects of Malat1 KO are more readily seen. The precise nature of how Nischarin leads to the enhancement of Malat1 levels is beyond the scope of this study. Nevertheless, our findings reveal that Nischarin expression status is a significant determinant of Malat1 expression and is an important consideration in experiments investigating cancer treatments which function by altering Malat1 function or expression.

## Materials and methods

### Cell culture and reagents

HEK-293T cells and MDA-MB-231 human breast cancer cell lines (a gift from Dr. Joan Massague, Memorial Sloan-Kettering Cancer Center, New York) were maintained in Dulbecco’s modified Eagle’s medium (DMEM) supplemented with 10% fetal bovine serum. 231-GFP control cells and 231-GFP-Nisch cells were previously created as described [14].

### LncRNA profiling

To focus on the most clinically relevant lncRNAs, the Human Disease-Related LncRNA Profiler (CAT# RA920D, System Biosciences) which is comprised of 83 lncRNAs chosen from the RNA database or the lncRNA database [15]. Total RNA was isolated from 231 and 231-Nisch cells treated with doxorubicin (doxo) at 1 mg/ml for 24hr then reverse transcribed with the RevertAidTM Reverse Transcriptase (Fermentas) and random hexamers (New England BioLabs). Values for the cells not receiving doxo treatment after normalization functioned as a basal expression level of loc285194; ΔΔCt values (ΔCT_no doxo_ - ΔCT_doxo_) were calculated to estimate relative fold change in expression as described [16].

### Generation of Malat1 KO cell lines

CRISPR/Cas9, dual gRNA, and donor vector expression plasmids were designed and produced as previously described [17]. We used 1μg DNA per 3.5cm well of CRISP/Cas9 constructs and transfected 231 cells using Opti-MEM and Lipofectamine 2000 as per the manufacturer's specifications (Invitrogen). HR210 was transfected into control cells to confer puromycin resistance in order to control for any effects resultant from the two week puromycin treatment period. After the selection, low-density plated cells were allowed to form individual colonies, which were scratched with a pipet tip and transferred to separate plates to ensure the monoclonality of each cell line.

### Normal and tumor breast tissues

Normal breast tissues and breast tumors tissue specimens were obtained as surgical samples from patients throughout the US. For procurement of these fresh frozen tissue sections, we collaborated with a consortium investigators who received breast samples from 5 divisions of the Cooperative Human Tissue Network: southern (Birmingham, AL), eastern (Philadelphia, PA), mid-Atlantic (Charlottesville, VA), Midwestern (Columbus, OH), and western (Nashville, TN). All specimens were kept at -80°C for long term storage.

### Reverse transcription and real time PCR

Malat1 and Nischarin expression in cell lines were examined in cell line samples as well as in human breast normal and tumor tissues by synthesizing cDNAs from 1 μg of tumor RNA using the high capacity cDNA reverse transcription kit (Applied Biosystems). GAPDH was used as a loading control. Primers for Nischarin and GAPDH control are described previously [14]. Malat1 and Nisch primers were designed using Primer3. Their sequences are as follows: Malat1 Fwd: 5’-GAC GGA GGT TGA GAT GAA GC-3’ and Malat1 Rvs: 5’-ATT CGG GGG TCT GTA GTC CT-3.’ Reactions were performed in triplicate. Each 20-μl PCR reaction volume included 2 μl of RT product, 1 μl primers, and 10 μl of SYBR Green I Master mix (Roche). The reactions were incubated in a 96 or 384-well plate at 95°C for 10 min, followed by 40 cycles of 95°C for 15 s and 60°C for 1 min. qPCRs were performed using a LightCycler480 Instrument (Roche). Human GAPDH was used as the housekeeping control to normalize Nisch and Malat2 expression. The ΔΔ*Ct* was calculated by subtracting the Δ*Ct* of the control cells from the Δ*Ct* of the experimental cells. Fold change was generated using the 2^−^ΔΔ^Ct^ equation.

Expression of miRNAs was quantitated using TaqMan microRNA assays (Applied Biosystems) specific for miR-17-5p and RNU6b control. Each sample was analyzed in triplicate. Reverse transcription was performed using the TaqMan MicroRNA Reverse Transcription Kit (Applied Biosystems), 10 ng of total RNA input. Real time PCR was performed using standard TaqMan protocols on a LightCycler480 Instrument (Roche). The 20-μl PCR reactions included 1.33 μl of RT product, 10 μl of TaqMan Universal PCR Master Mix, No AmpErase UNG (Applied Biosystems), and 1 μl of primer and probe mix (Applied Biosystems). The reactions were incubated in a 96-well plate at 95°C for 10 min, followed by 40 cycles of 95°C for 15 s and 60°C for 1 min. The level of miRNA expression was measured using *Ct* (threshold cycle). The Δ*Ct* was calculated by subtracting the *Ct*_U6_ from the *Ct*_miR-17-5p_. The ΔΔ*Ct* was calculated as described above.

### MTT proliferation assays

Cell proliferation was assessed using a 3-(4,5-dimethylthiazol-2-yl)-2,5-diphenyltetrazolium bromide (MTT) assay. Cells were seeded at 5,000/well in 96 well plates and incubated for 1–4 days, after which 10 μl of 5 mg/ml MTT (Cayman Chemical) was added to each well. The wells were then incubated at 37°C for 3.5 hr. The purple-blue MTT formazan precipitate was dissolved in 150 μl of MTT solvent (4 mM HCl, 0.1% NP-40 in isopropanol). Increases in cell number result in greater amounts of formazan production and thus increased optical density, measured at 562nm in a micro-plate reader (Bio-Rad). Since a uniform cell number was plated initially, increased absorbance in one sample compared to another indicates a greater rate of cell proliferation was present.

### Wound healing assays

231 cells were plated into 3.5cm well plates until near-confluency. We then scratched across the center of each well using a pipet tip to create a gap into which the cells could migrate. Images were captured at time zero, 12hr, 24hr and 48hr post-scratch using a Nikon Eclipse Ti-S microscope.

### Migration assays

1x10^6^ 231 cells in serum-free DMEM were seeded into the fibronectin-coated (upper chambers) of 24-well Transwell plates with 8.0μm polycarbonate membrane inserts (Corning). Complete DMEM (containing 20% FBS) was placed in the lower chamber and served as a chemoattractant. A replicate of each using serum free medium (SFM) in the lower chamber was used as a control for random migration. The cells on the upper surface of the filter were removed by a cotton swab after 18 hours. Migrated cells were fixed in 4% paraformaldehyde and stained with crystal violet, and were visualized by microscope. Randomly selected fields of uniform size were chosen and the cells in each were manually counted. At least three different fields were counted for each, and each experiment was performed in triplicate.

### Statistical analyses and datamining

Results from experiments are expressed as means ± SEM. In making comparisons between two groups for statistical analyses in all experiments, we used two-tailed nonpaired *t*-test (GraphPad Prism, version 5). Differences with p values of <0.05 were considered significant. The UALCAN web-based resource was utilized to analyze the TCGA data and generate graphical outputs of cancer gene transcriptomics and patient survival information (http://ualcan.path.uab.edu/). We used this resource to compare Nischarin andMalat1 expression between cancer subtypes. For XY correlation plots, we retrieved Nischarin and Malat1 expression data from the GTEx portal database (gtexportal.org) which contains gene expression across a panel of 52 normal tissue types. The expression level of Malat1 and Nisch in each individual tissue type was imputed as a single data point with X and Y coordinates in a correlation plot. Spearman correlation r was calculated for the 52 XY pairs in the plot and fitted with a trend line to display the correlation using GraphPad Prism. The Oncomine platform (Oncomine.org) was used to access the Curtis Breast Statistics database and analyze both gene expression of nischarin and Malat1 in human breast cancer samples according to their subtypes (invasive ductal and invasive lobular). Images of summary output data were captured and shown as they appeared in the Oncomine interface. Survival curves for Nisch and Malat1 were generating using KM plotter (kmplot.com).

## Results

### Expression of multiple lncRNAs is altered in Nischarin-low versus Nischarin overexpressing cells

**Fig 1:**
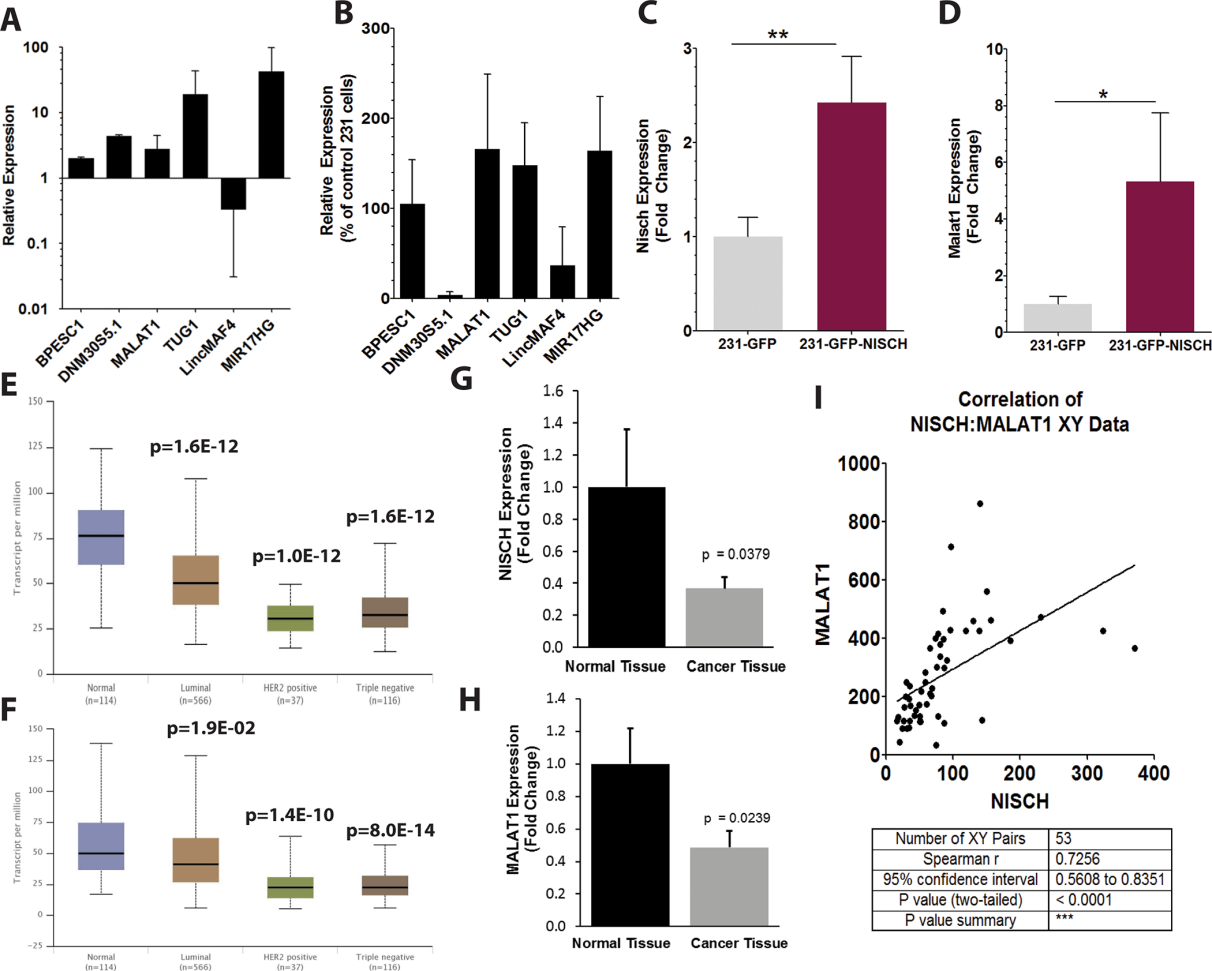
Expression of multiple lncRNAs is altered in Nischarin overexpressing cells. (A and B) Microarray of 231-GFP and 231-GFP Nisch cells evaluating lncRNAs deviating the most between the two cell lines. qPCR of Nisch (C) and Malat1 (D) expression in 231-GFP and 231-GFP-Nisch cells. Expression of Nischarin (E) and Malat1 (F) in normal breast and various breast cancer subtypes retrieved from the TGCA accessed using UALCAN. Expression of Malat1 (G) and Malat1 (H) in a panel of normal and cancer human breast tissues as measured by qPCR assay. Data are shown as relative fold change (n = 8 for normal tissues and n = 15 for tumor samples). (I) XY correlation plot showing the positive relationship between Nisch and Malat1 across a panel of 55 different tissue samples accessed using the NCBI GTEx database.

We initiated the project by employing a microarray for 83 lncRNAs transcripts to screen for changes in lncRNAs expression between samples, which we previously described [17]. Total RNA from MDA-MB-231 breast cancer cells and 231 cells overexpressing Nischarin (231-Nisch) was collected and analyzed using this lncRNAs-specific microarray to identify lncRNAs which are most significantly altered between the two cell types. Of those enhanced in cells with ectopic Nischarin, MIR17HG showed the largest change, followed by several others, including TUG1 DNM30S5.1, MALAT1, BPESC1. (**Fig 1A**). The microarray was duplicated to confirm the pattern of lncRNA expression (**Fig 1B**). Once again, MIR17HG, MALAT1, TUG1 and BPESC1 showed strong increases, however, the increase in DNM30S5.1 was not reproduced. The increase we observed in MIR17HG was supported by evidence that the miRNA derived from this host gene, miR-17-5p was also found to be elevated (**S1 Fig**). We ultimately chose to focus on Malat1 in subsequent experiments, primarily because of its important role in cancer, the precise nature of which remains a controversial subject. To confirm the microarray data for Malat1, we performed qRT-PCR for Nischarin and Malat1. As anticipated, the 231-Nisch cells greater Nischarin expression (**Fig 1C**), while also showing a several fold increase in MALAT1 expression in the 231-Nisch cells over control (**Fig 1D**). We also noted a similar pattern of expression between Nisch and Malat1 in normal breast tissue and various breast cancer subtypes in TCGA expression data accessed using UALCAN online data portal (http://ualcan.path.uab.edu/). For both genes, expression was highest in normal breast, with the TNBC subtype showed the lowest expression (**Fig 1E and 1F**). Notably, the 231 breast cancer cell line is a prototypical tissue culture model for TNBC. The observation that this type of cancer exhibits low levels of Malat1 in human samples as well signals the potential usefulness of using this cell line to study Malat1 in our Nisch-dependent context.

In support of this TCGA analysis, we were able to reproduce the findings in an independent set of normal and tumor breast tissues preciously assembled by our lab. qPCR analysis showed statistically significant reductions in both Malat1 and Nisch, with Nischarin showing the greater reduction in magnitude of the two (**Fig 1G and 1H**). Moreover, the positive association between Nischarin and Malat1 expression is seen across a broad variety of tissue types using an approach to compare Nisch and Malat1 expression data devised by our lab. We first accessed data from the Broad Institute’s GTEx portal to browse RNA-seq data for genes of interest across a panel of 52 human tissue types.(S2 Fig). By imputing the mean expression level of Nisch on the X axis and Malat1 on the Y axis from each tissue into a correlation plot, we found a significant positive correlation across the dataset (**Fig 1I**).

### Generation of Malat1 KO of 231 cells in a background of high and low Nischarin expression

**Fig 2:**
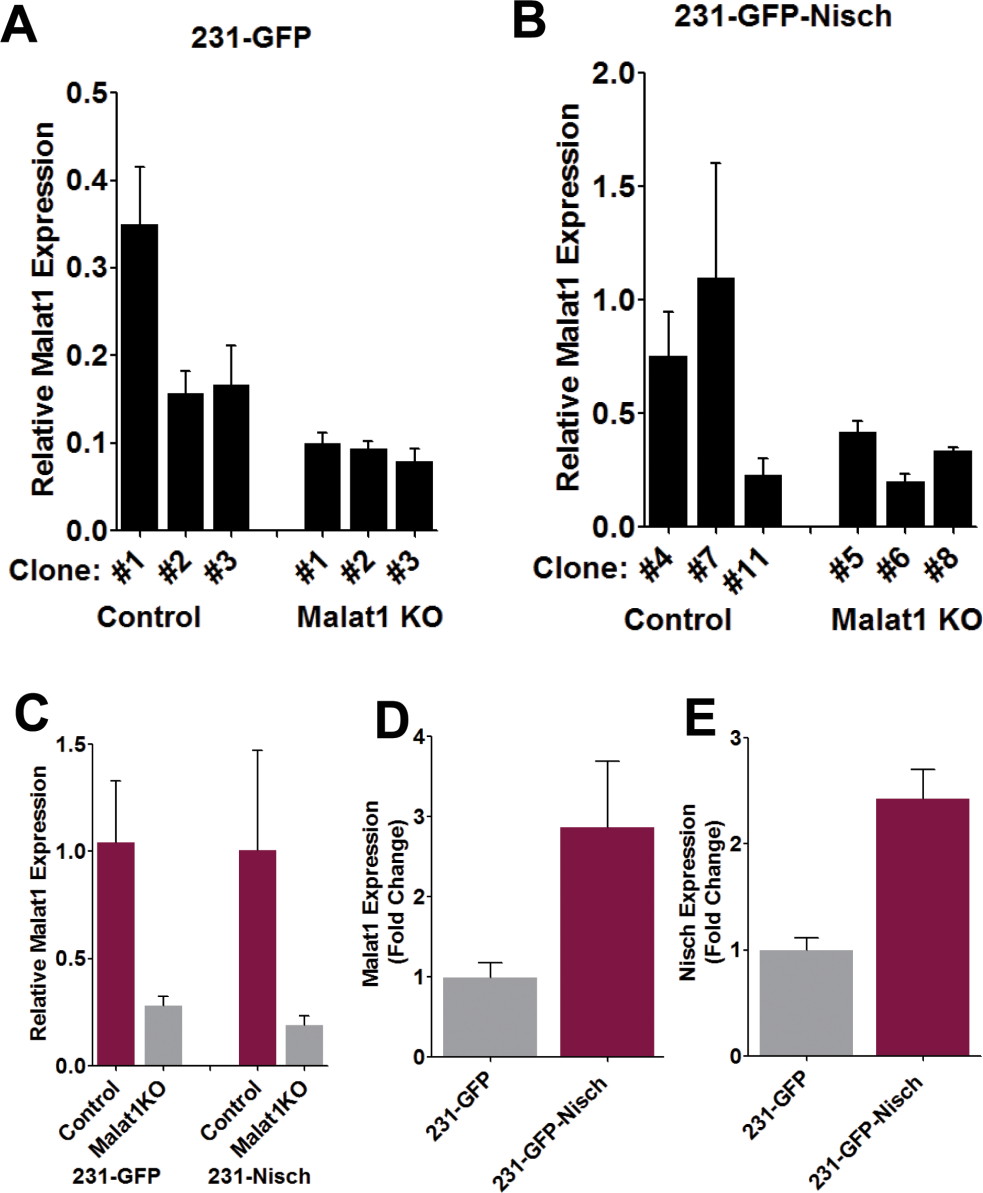
Generation of Malat1 KO of 231 cells in a background of high and low Nischarin expression. (A and B) Malat1 expression following Malat1 KO in both 231-GFP and 231-GFP-Nisch cell clones. (C) Expression of Malat1 in the four selected clones: 231-GFP control #1, 231-GFP Malat1 KO #3. 231-Nisch control #7, and 231-Nisch Malat1 KO#6. Fold change in Malat1 (D) and Nisch (E) following puromycin selection between the 231-GFP and 231-Nisch control cells (without malat1 KO), confirm the 231-Nisch cells maintained the comparative increased expression levels of these genes following clonal selection and expansion.

Next, we wished to evaluate the effects of Malat1 knockout from 231 cells with and without Nisch expression. Thus, we utilized a CRISPR/Cas9 system as described in a previous publication [17]. Following puromycin selection, several colonies of each were selected and assessed for the degree of Malat1 KO (**Fig 2A and 2B**). However, as the 231-GFP cells already displayed attenuated Malat1 expression level from the start, the absolute decrease was less pronounced in 231- cells, since the gene is already expressed at low levels (**Fig 2A**). In contrast, KO of Malat1 in the 231-Nisch cells (which exhibit higher Malat1 expression) led to a greater change in magnitude, but overall the fold change of Malat1 expression in resulting cells was roughly comparable. A single KO clone with the greatest degree of Malat1 reduction was chosen for each and used in the ensuing experiments (**Fig 2C**). In both of the KO cell lines, the level of Malat1 expression was reduced to roughly 20% of the control cells, which is similar to that produced in previous experiments which utilized this system [17].To confirm the process of transfecting and puromycin selection did not affect the pattern of Nischarin expression in the resultant cells, we collected RNA from the four chosen clones and performed qPCR analysis which confirmed the predicted expression status of Malat1 and Nisch in each of the 4 cell lines (**Fig 2D and 2E**).

### Malat1 KO enhances cell proliferation and migration in a Nischarin-dependent context

**Fig 3:**
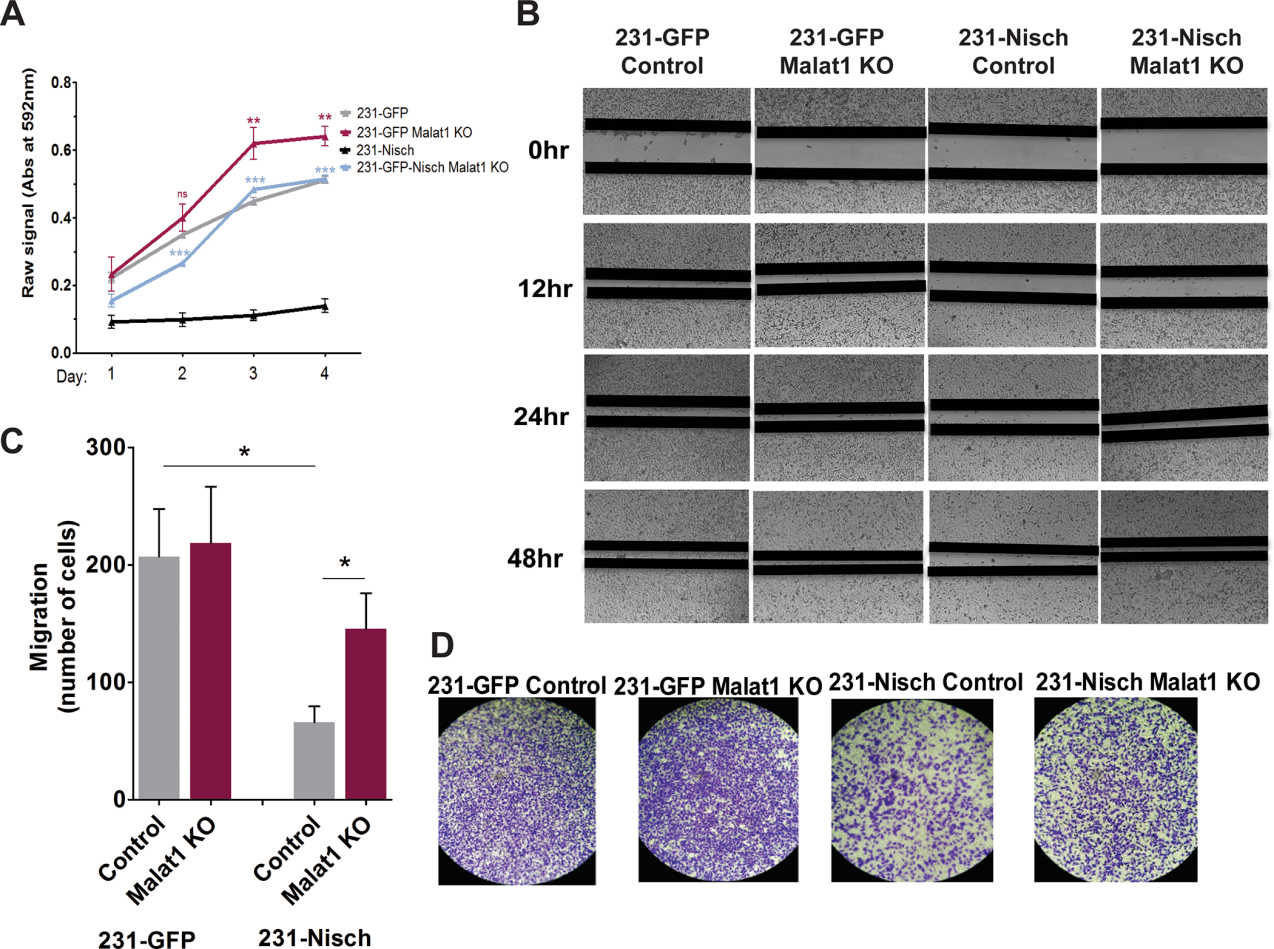
Malat1 KO enhances cell proliferation and migration in a Nischarin -dependent context. (A) Timecourse MTT assay of the four chosen 231 cell clones showing the number of viable cells thought the 96 hour incubation. (B) Representative images of wound-healing assays in the four 231 cell lines at times 0hr, 12hr, 24hr, and 48hr post scratch. (C and D) Transwell migration assay data and representative images of each of the cell lines after 24 hour incubation.

We next sought to assess what functional consequences Malat1 KO might have in these cells and whether any changes observed occur in a Nischarin-dependent context. We began by evaluating the proliferative capacity using a time course MTT assay of the four cell lines over several days. The loss of Malat1 led to an increase in cell growth compared to control in both the control (Nisch-low) and Nisch overexpressing cell lines (**Fig 3A**). However, interestingly, the effects of Malat1 KO was far more pronounced in the 231-Nisch cells than in the 231 control cells, which have low Nisch expression. To assess whether Malat1KO could affect cell migration, we employed a wound-healing assay. Analogous to the MTT assay, cells lacking Nisch had greater capacity to fill the wound over the duration of the experiment (**Fig B**). In cells with ectopic Nisch expression, migration was more gradual; however, Malat1 KO in these cells greatly restored migration, resembling that of the 231-Nisch deficient cells. As another measure of estimating migration, we performed a Transwell migration assay, which showed similar findings to the wound-healing assay (**Fig 3C and 3D**). Once again, Malat1 KO had the most pronounced effect in the context of Nisch-high cells, whereas it had no observable effects in a Nisch-low context.

### Experimental study findings are supported by expression and survival data acquired from online databases

**Fig 4:**
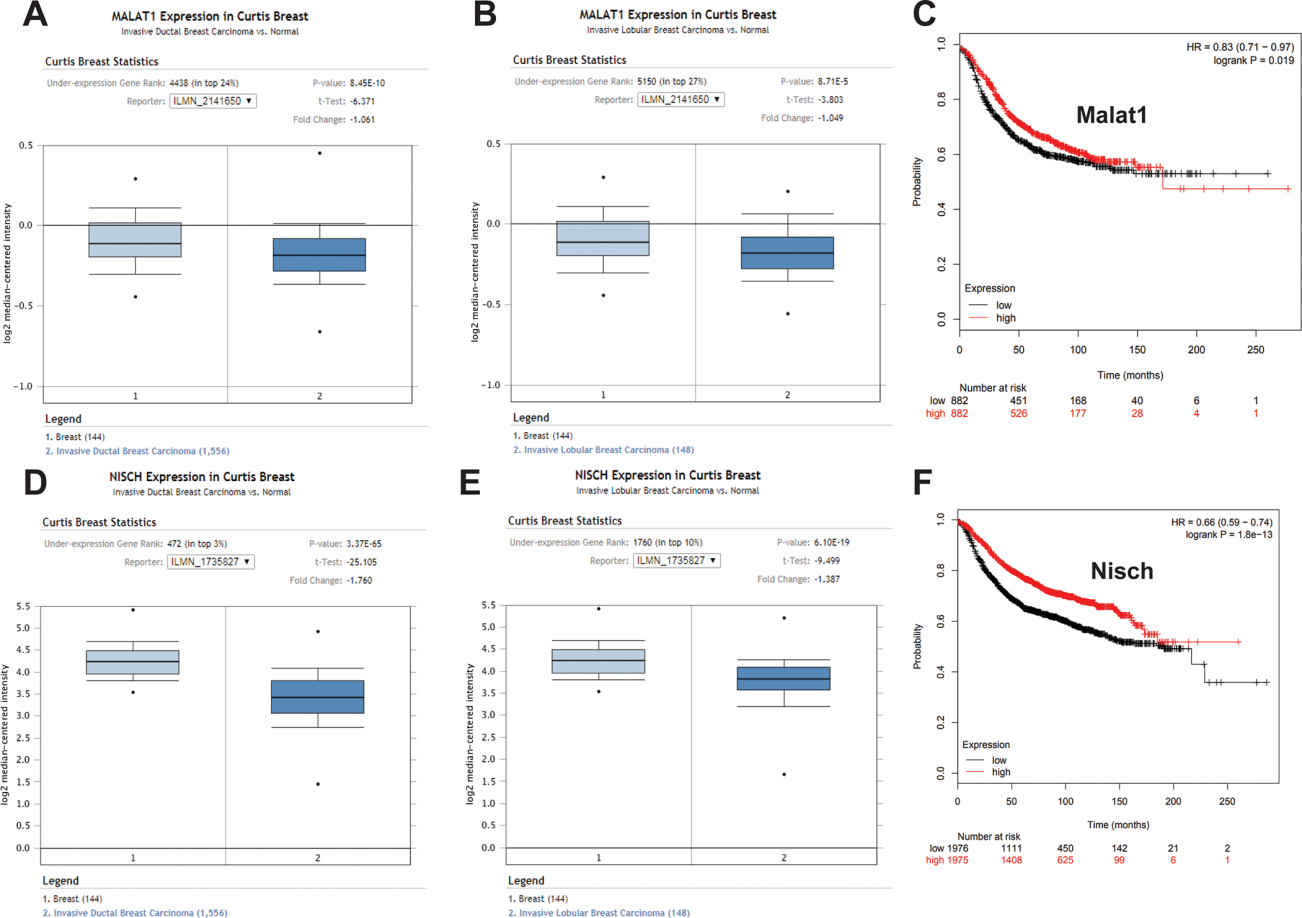
Archival database findings for Malat1 expression and outcomes correspond with study experimental findings. Curtis Breast Cancer statistics were accessed using the Oncomine platform for Malat1 in ductal (A) and lobular (B) breast carcinoma. (C) Survival curve for patients with high or low Malat1 expression using KMplot.com. Curtis Breast Cancer statistics were accessed as before for Nischarin expression in ductal (D) and lobular (E) breast carcinoma. (F) Survival curve for patients with high or low Nisch expression using KMplot.com.

Using publically available online data resources, we sought to compare our findings in cell culture with data from human breast cancer subjects to identify any correlations. Since both Nisch and Malat1 appear to confer an inhibitory effect upon cell growth and migration in breast cancer, we expected these genes to be reduced in breast tumors compared to normal tissue controls. Through the Oncomine data portal (Oncomine.org) we found support for this hypothesis, which indicated Malat1 is reduced in both ductal and lobular breast cancer morphological subtypes compared to control normal breast tissue (**Fig 4A and 4B**). Nischarin was also found to be reduced in both ductal and lobular subtypes (**Fig 4D and 4E**). Moreover, increased levels of both Malat1 (**Fig 4C**) and Nischarin (**Fig 4F**) were found to positively correlate with enhanced patient recurrence-free survival.

## Discussion

By using a broad-based, nonbiased approach in our initial screening experiments to identify the most significantly altered lncRNAs by the presence or absence of Nischarin expression we were able to pursue a course of experimental inquiry in an impartial manner from the onset of the project. This strategy enabled us to focus solely on those lncRNAs which show the most promise as Nischarin interacting partners, whether directly or indirectly.

While the precise role of Malat1 in cancer is still not fully understood, a number of studies indicate that Malat1 frequently functions as an oncogene and is associated with metastasis and poor prognosis [18–24]. However, the work we present here supports the notion that it functions as a contextual tumor suppressor gene, specifically, in breast cancers expressing sufficient levels of Nischarin. The evidence that Malat1 is a tumor suppressor in breast cancer is seemingly contrary to many studies investigating its functions in other types of cancer, such as lung, in which it was first identified and subsequently titled accordingly (“Metastasis-Associated Lung Adenocarcinoma Transcript 1”) [5]. However, in breast cancer, the function of Malat1 is currently in dispute; recent studies have supplied evidence describing potential mechanisms for Malat1 to function as a tumor suppressor gene by inhibiting EMT through suppression of the PI3K-AKT pathway [11], inactivation of ERK/MAPK signaling [25], or dampening CD133 expression [26]. Nevertheless, other studies have reported alternative findings, wherein Malat1 is associated with greater cell migration, invasion and metastasis [12,27,28]. Notably, these findings appear at odds with human breast cancer data from the TCGA, which suggests that Malat1 expression is reduced in breast cancer compared to normal tissue, particularly in the triple negative subtype (**Fig 1**) and, moreover, that higher Malat1 expression corresponds with improved recurrence free survival (**Fig 4**). Data from the Curtis Breast Statistics database similarly indicate that Malat1 tends to be reduced in breast cancer tissues versus control (**Fig 4**).

A simple means of reconciling these discrepancies is not readily apparent. One possible explanation however, may be the drawn by considering the importance of context-specific binding partners in determining Malat1’s function. The functionality of lncRNAs often depends on their ability to form complexes with proteins or other RNA species, which then together execute their regulatory function on gene expression. In this way, the effect of expressing a lncRNA can differ widely depending on what binding partners are available for cooperation within the cell. Thus, in one cell type, a lncRNA may associate with a protein (or set of proteins) that results in the formation of a complex that enhances oncogenic gene expression and cell function, while the very same lncRNA, when expressed in another cell line with entirely different protein binding partners, could produce the opposite effect. Unfortunately, the field of lncRNA is still comparatively new, and thus, a thorough appreciation of lncRNAs and their numerous interactions and functions has not yet been realized.

In addition to our Malat1 findings, the other component of this study involves unraveling how Nischarin is involved with Malat1 expression and function. For reasons that are still unclear, the elevation of Nischarin is matched by a concordant increase in Malat1 expression. However, a precise accounting of the molecular mechanism underpinning Nischarin’s capacity to enhance Malat1 expression is beyond the scope of this study. Nevertheless, this observation can be of benefit for ongoing studies of the Malat1 lncRNA, since awareness of a Nischarin expression status is relevant in predicting the level of Malat1 that is likely to be present in cell models.

## Supporting information

S1 Fig Increased expression of miR-17-5p correlates with increased expression of its host gene, MIR17HG. TaqMan qRT-PCR for miR17-5p in 231-GFP and 231-GFP-Nisch breast cancer cells.
(TIF)

S2 Fig Malat1 expression patterns correlate with Nischarin in normal human tissues. The GTEx database was accessed to analyze an array of 52 normal tissue types for their Nischarin and Malat1 expression patterns, which we plotted against one another to illustrate the tendency for the expression of these two genes to correlate positively with one another across the panel of tissues.
(TIF)
