# Supplementary figures and images for: Expression of long noncoding RNA MALAT1 correlates with increased levels of Nischarin and inhibits oncogenic cell functions in breast cancer
Source: PLoS One. 2018 Jun 18;13(6):e0198945. doi: 10.1371/journal.pone.0198945 (PMC6005468; doi:10.1371/journal.pone.0198945)

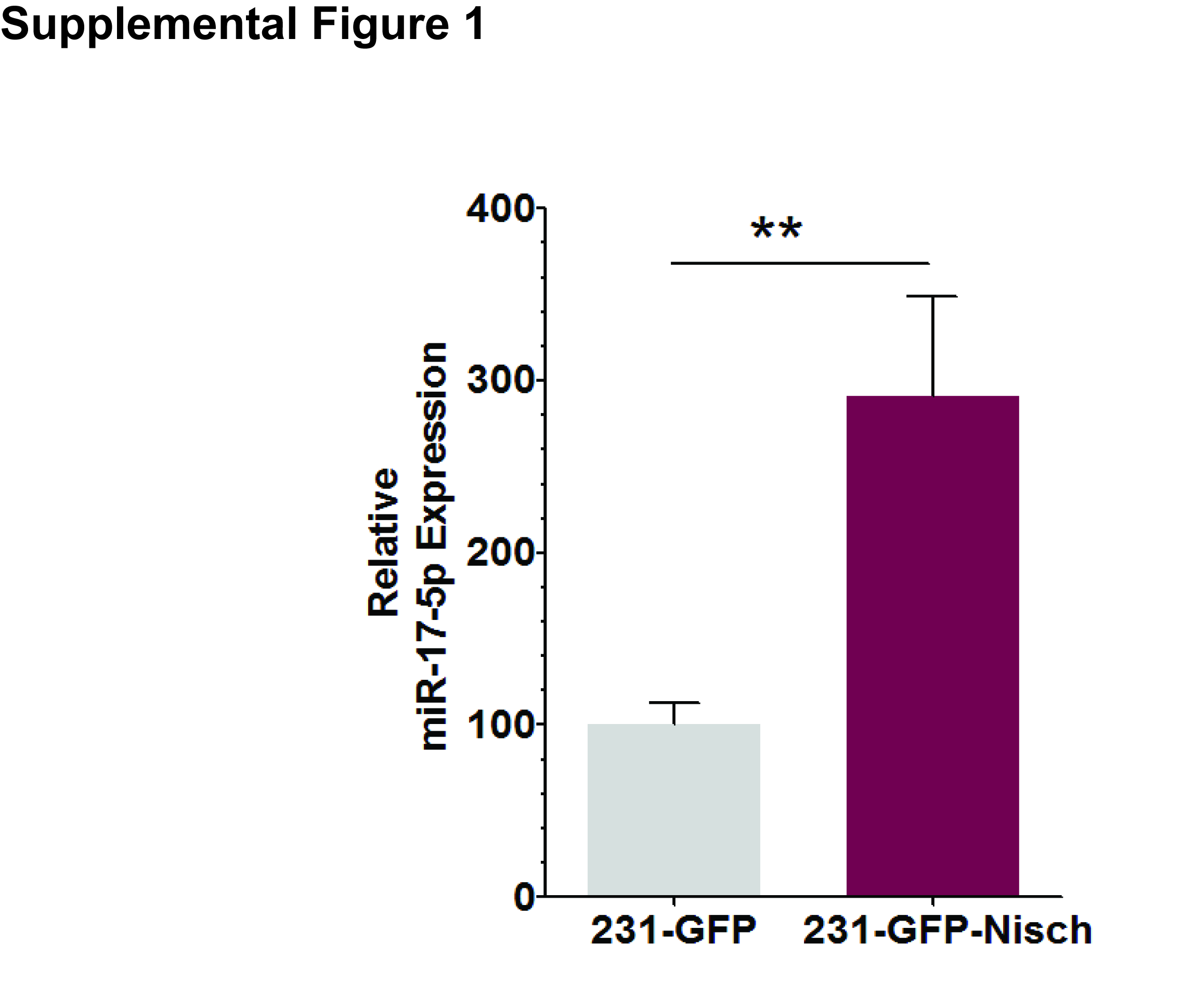

Supplement: S1 Fig — TaqMan qRT-PCR for miR17-5p in 231-GFP and 231-GFP-Nisch breast cancer cells. (TIF) [file pone.0198945.s001.tif]

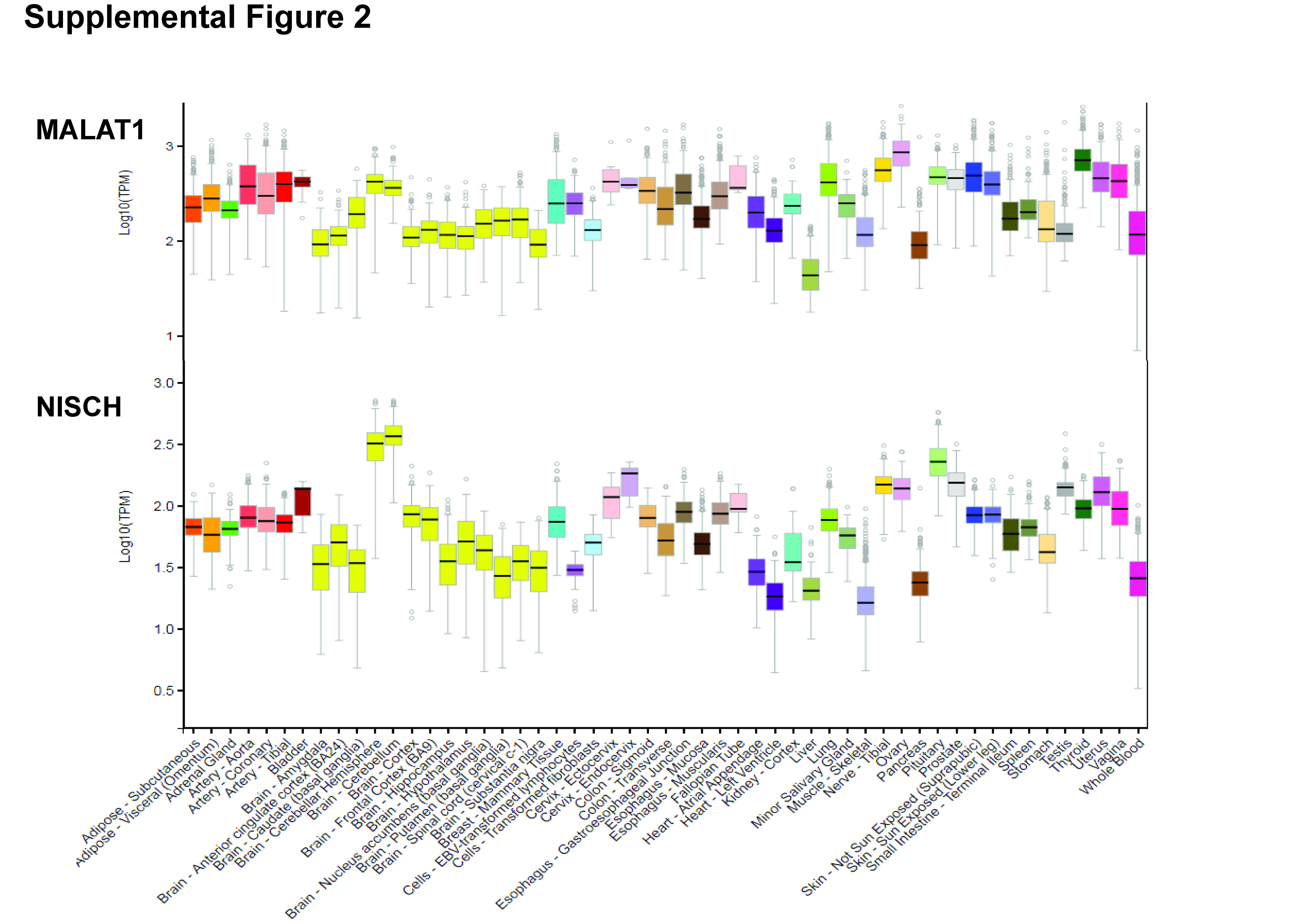

Supplement: S2 Fig — The GTEx database was accessed to analyze an array of 52 normal tissue types for their Nischarin and Malat1 expression patterns, which we plotted against one another to illustrate the tendency for the expression of these two genes to correlate positively with one another across the panel of tissues. (TIF) [file pone.0198945.s002.tif]
